# Expression of microRNA in follicular fluid in women with and without PCOS

**DOI:** 10.1038/s41598-019-52856-5

**Published:** 2019-11-08

**Authors:** Alexandra E. Butler, Vimal Ramachandran, Shahina Hayat, Soha R. Dargham, Thomas Keith Cunningham, Manasi Benurwar, Thozhukat Sathyapalan, S. Hani Najafi-Shoushtari, Stephen L. Atkin

**Affiliations:** 1 Diabetes Research Center (DRC), Qatar Biomedical Research Institute (QBRI), Hamad Bin Khalifa University (HBKU), Qatar Foundation (QF), PO Box 34110 Doha, Qatar; 2 Weill Cornell Medical College Qatar, Education City, PO Box 24144 Doha, Qatar; 3 Academic Diabetes, Endocrinology and Metabolism, Hull York Medical School, Hull, UK; 4 Royal College of Surgeons in Ireland Bahrain, Bahrain, Bahrain

**Keywords:** Reproductive biology, Reproductive disorders

## Abstract

Several studies have shown the expression of small non-coding microRNA (miRNA) changes in PCOS and their expression in follicular fluid has been described, though the number of studies remains small. In this prospective cohort study, miRNA were measured using quantitative polymerase chain reaction (qPCR) in 29 weight and aged matched anovulatory women with PCOS and 30 women without from follicular fluid taken at the time of oocyte retrieval who were undergoing *in vitro* fertilization **(**IVF); miRNA levels were determined from a miRNA data set. 176 miRNA were detected, of which 29 differed significantly between normal women and PCOS women. Of these, the top 7 (p < 0.015) were miR-381-3p, miR-199b-5p, miR-93-3p, miR-361-3p, miR-127-3p, miR-382-5p, miR-425-3p. In PCOS, miR-382-5p correlated with age and free androgen index (FAI), miR-199b-5p correlated with anti-mullerian hormone (AMH) and miR-93-3p correlated with C-reactive protein (CRP). In normal controls, miR-127-3p, miR-382-5p and miR-425-3p correlated with the fertilisation rate; miR-127-3p correlated with insulin resistance and miR-381-3p correlated with FAI. Ingenuity pathway assessment revealed that 12 of the significantly altered miRNA related to reproductive pathways, 12 miRNA related to the inflammatory disease pathway and 6 were implicated in benign pelvic disease. MiRNAs differed in the follicular fluid between PCOS and normal control women, correlating with age, FAI, inflammation and AMH in PCOS, and with BMI, fertilization rate (3 miRNA), insulin resistance, FAI and inflammation in control women, according to Ingenuity Pathway Analysis.

## Introduction

Polycystic ovarian syndrome (PCOS) affects 9-21% of the female population and results in anovulatory infertility^1^, and is associated with clinical and biochemical hyperandrogenism, and insulin resistance (IR), hypercholesterolemia and type 2 diabetes^2^. The underlying pathophysiology of PCOS is unclear and complex metabolic pathways underlying PCOS, involving deregulated carbohydrate, fat and protein metabolism, have been described^3^. A single unifying mechanism to account for these disparate physiological and clinical observations has not been described, though noncoding RNA expression regulating gene expression may be a candidate^4^.

MicroRNAs (miRNAs) are small noncoding single-stranded RNA molecules 18-24 nucleotides in length that directly regulate gene expression post-transcriptionally. Canonical miRNA binding occurs at the 3’ untranslated regions of the target messenger RNAs (mRNAs), that leads to the repression of mRNA expression resulting in protein translation inhibition. Several critical regulatory functions common to many biological processes are regulated by miRNAs that include cell growth and turnover, and the stress response^5^. The miRBase miRNA registry now includes over 2600 mature human miRNAs, of which only approximately 600 are confidentially annotated and can be considered as validated microRNAs^6^. A single miRNA may modulate the expression and function of hundreds of downstream target genes^7^. Amplification or inhibition of the miRNA signal through feedback on gene regulation may cause dramatic deregulation of physiologic cellular functions by just a few miRNAs, such as that seen following obesity surgery^8^. There is increasing evidence showing the involvement of miRNA in the pathogenesis of both type 1 and type 2 diabetes, and their identification as novel disease biomarkers^9^. Circulating miRNA have been described in PCOS^10–15^ and the expression of 3 miRNAs have been suggested to have utility as PCOS biomarkers^10^. However, whilst miRNA-21, miRNA-27b, miRNA-103, and miRNA-155 have been reported to be differentially expressed in obesity and in PCOS^13^, their regulation is likely to be complex.

The intrafollicular environment is critical for oocyte development, maturation and quality; constituents of the follicular fluid reflect local secretory activity of the oocyte, granulosa and theca cells^16^. Studies on miRNAs in follicular fluid are limited and conflicting with, for example, one study finding miR-132 and miR-320 expression reduced in women with PCOS^17^, another finding miR-320 expression increased in PCOS^18^ and a third reporting no change in miR-320a in PCOS^19^. Another study found five miRNAs (miR-9, miR-18b, miR-32, miR-34c and miR-135a) to have increased expression in women with PCOS^12^. When PCOS was phenotypically stratified, decreased levels of miR-24-3p, miR-29a, miR-151-3p and miR-574-3p were found in PCOS women versus controls, and differentially high expression of miR-518f-3p was found in hyperandrogenic versus normo-androgenic PCOS women^20^.

Given the differing results of miRNA expression reported that could reflect PCOS alone, obesity, insulin resistance, androgen levels and/or a metabolic phenotype among other factors, this study was undertaken to assess miRNA in the follicular fluid in age and BMI matched women with and without PCOS who underwent the same IVF protocol for subfertility.

## Materials and Methods

“This prospective cohort study was performed from January 2014 to January 2016 within the Hull IVF Unit, UK following approval by the Yorkshire and the Humber NRES ethical committee, UK and all patients gave their written informed consent. Subject demographics are shown in Table 1. The PCOS subjects were recruited using the revised 2003 criteria^21^, namely any 2 out of 3 criteria were met; menstrual disturbance (oligo or amenorrhoea), clinical and/or biochemical signs of androgenism and polycystic ovaries on ultrasound, with the exclusion of other conditions. All women were on folic acid 400 mcg daily but not any other medication. Exclusion criteria were patients with diabetes, renal or liver insufficiency, acute or chronic infections, systemic inflammatory diseases, age <20 or >45 years, or having known immunological disease. Liver ultrasound was performed at the same time to exclude non alcoholic fatty liver disease. Study participants had no concurrent illness and were not on any medication for the preceding nine months except study medications. None of the patients had had a successful pregnancy or miscarriage in at least five years prior to study entry. Diabetes was excluded by a 75 g oral glucose tolerance test. Non-classical 21-hydroxylase deficiency, hyperprolactinaemia, Cushing’s disease and androgen-secreting tumours were excluded by appropriate tests”^22^.

**Table 1:** Mean Demographic and biochemical data.

	Control (n = 30)	PCOS (n = 29)	p-value
	Mean data ( ± SD)	Mean data ( ± SD)	
Age	32.6 ± 4.7	30.9 ± 4.8	0.14
BMI	25.5 ± 3.6	26.0 ± 3.8	0.56
Menarche	13.0 ± 2.0	13.0 ± 1.1	0.99
Total Antral Follicle Count	17.2 ± 6.8	38.4 ± 17.8	0.0001^*^
Testosterone (nmol/l)	0.8 ± 0.4	1.4 ± 0.8	0.0004^*^
Free androgen index	1.35 ± 0.6	4.21 ± 2.9	0.0001^*^
Fasting Insulin (mIU/ml)	7.68 ± 4.0	8.13 ± 4.7	0.69
Fasting Glucose (mmol/L)	4.81 ± 0.4	4.62 ± 0.4	0.06
HOMA-IR	1.71 ± 1.0	1.72 ± 1.0	0.97
SHBG (mmol/l)	110.9 ± 82.4	63.9 ± 49.8	0.01^*^
AMH (pg/ml)	21.9 ± 10.4	55.8 ± 14.5	0.001^*^

BMI = body mass index; HOMA-IR = insulin resistance; SHBG = sex hormone binding globulin; AMH = antimullerian hormone. * = statistically significant result.

### Sample Collection

“All patients underwent a standard IVF antagonist protocol. The patients commenced their rFSH stimulation on day 2 of their menstrual cycle using either Merional (Pharmasure) or Gonal-F (Merck Serono). A GnRH antagonist (Cetrotide: Merck Serono) was used to prevent a premature LH surge^22^”.

“The patients underwent ultrasound scans from day 7 to observe the ovarian response to stimulation and these were repeated every 48 hours. The scans were used to measure the diameters of the follicles, thus observing response and follicle numbers. Final maturation was triggered when two or more leading follicles were ≥18 mm using human chorionic gonadotrophin (hCG, Pregnyl (Merck Sharp and Dohme). Follicular fluid was collected at the same time as oocyte harvesting, was centrifuged at 3500 g for 15 minutes and placed into aliquots and frozen at −80 °C until analysis^22^”.

“Transcervical embryo transfer was performed and embryos were classified using standard criteria^23^ at the cleavage stage (day 2 to 3 after egg collection) and for blastocyst stage (day 5 to 6 after egg collection). Top Quality embryos were graded on Day 3 as per Alpha Consensus^24^. Embryo transfers were performed on either day 3 or ideally at day 5 (blastocyst) to give the best chance for implantation, as this timing is similar when compared to natural cycle embryos moving into the uterus”^22^.

“A fasting blood sample was taken on day 21of the luteal phase of the cycle before commencing IVF treatment, and centrifuged at 3500 g for 15 minutes and placed into aliquots and frozen at −80 °C until analysis. All PCOS women were oligomenorrheic, none having had a period within the preceding 6 weeks. Serum and plasma was stored frozen at −80 °C pending analysis. Serum testosterone was measured by isotope dilution liquid chromatography- tandem mass spectrometry (Waters Corporation, Manchester, UK). Sex hormone binding globulin (SHBG) was determined by an immunometric assay with fluorescence detection on the DPC Immulite 2000 analyzer using the manufacturer’s recommended protocol. The free androgen index was obtained as the total testosterone x100/SHBG. Serum insulin was assayed using a competitive chemiluminescent immunoassay performed on the manufacturer’s DPC Immulite 2000 analyzer (Euro/DPC, Llanberis, UK). The analytical sensitivity of the insulin assay was 2 µU/ml, the coefficient of variation was 6%, and there was no stated cross-reactivity with proinsulin. Plasma glucose was measured using a Synchron LX 20 analyzer (Beckman-Coulter), using the manufacturer’s recommended protocol. The coefficient of variation for the assay was 1.2% at a mean glucose value of 5.3 mmol/liter during the study period. The insulin resistance was calculated using the HOMA method [HOMA-IR = (insulin × glucose)/22.5], and pancreatic beta cell sensitivity measured by HOMA-β [HOMA-β = (20 × insulin)/glucose −3.5]^22^”.

## MicroRNA analysis

“Currently, due to accuracy, simplicity, and greater reproducibility, quantitative polymerase chain reaction (qPCR) is the favored method for determining microRNA expression when compared to other hybridization or sequencing-based technologies^25^. MicroRNA was isolated from 200 µl EDTA plasma using the miRCURY™ RNA Isolation Kit (Exiqon A/S, Denmark) following the manufacturer’s instructions and applying an RNA Spike-in kit (Exiqon A/S, Denmark) for assessment of hemolysis and contamination with blood cells and RNA isolation efficiency. The yield of microRNA preparation from plasma samples was monitored by including carrier RNA from the bacteriophage MS2. The quality and the integrity of the RNA were assessed by a 2100 Bioanalyzer instrument (Agilent Technologies), equipped with the small RNA specific chip. The expression of microRNA levels was retrieved from a microRNA data set generated by RNA sequencing of more than 390 different human tissue and primary cell samples^8,26^”

## Statistics

There was no information regarding changes in miR on which to base a sample size calculation. For such pilot studies, Birkett and Day^27^ suggest a minimum of 20 degrees of freedom to estimate variance from which a larger trial could be powered, hence 24 subjects in each group were recruited. Statistical analysis was performed using SPSS (v22, Chicago, Illinois). Descriptive data is presented as mean ± SD for continuous data and n (%) for categorical data. T-tests or Mann Whitney tests were used to compare means/medians where appropriate. Linear associations were assessed using the Spearman’s correlation test. Mean normalization was performed before analysis using the global mean of each miRNA. Using GenEx software that accompanied the Bioanalyzer, normalization was performed to achieve a global mean of all miRNAs with Ct below 35. An unpaired t-test was used to test the paired changes between PCOS and controls in miRNA levels. False discovery rates (FDR) of q < 0.05^28^ were considered significant. Pathway analysis was performed using Ingenuity Pathway Analysis (Qiagen), and those pathways over-represented by the FDR-significant miRNA changes at q < 0.05^28^ were identified.

## Results

Baseline characteristics of the 59 patients, 29 PCOS and 30 normal controls, are shown in Table 1 where it can be seen that patients were non-obese, age and weight matched and did not differ in their insulin resistance. There were significant differences in ovarian reserve parameters antral follicle count (AFC) and anti-Mullerian Hormone, (AMH), and androgen status between the groups; however, there was no significant difference in fasting insulin or HOMA-IR (Table 1).

IVF parameters are shown in Table 2, where it can be seen that more follicles were aspirated and eggs retrieved (p < 0.05) and the fertilization rate was higher for PCOS (p < 0.0003).

**Table 2:** Mean outcome data for stimulated ovarian cycle for Control and PCOS groups. G3D3: Top Quality embryos on Day 3 as per Alpha Consensus^24^. * = statistically significant result.

	Control (N = 30)	PCOS (N = 29)	p-value
	Mean ( ± S.D.)	Mean ( ± S.D.)	
Endometrium at oocyte retrieval (mm)	10.31 ± 1.78	10.72 ± 2.06	0.42
Follicles aspirated	11.47 ± 5.11	15.96 ± 5.30	0.002^**^
Eggs Retrieved	8.47 ± 5.08	11.29 ± 5.02	0.04^*^
Fertilisation rate	4.82 ± 2.65	8.43 ± 3.87	0.0003^***^
Cleavage	4.68 ± 2.72	7.26 ± 4.40	0.01^*^
G3D3	3.00 ± 2.29	4.17 ± 3.47	0.16
Blastocyst	1.46 ± 1.77	2.91 ± 3.01	0.05^*^
Clinical Pregnancy	10	7	0.24

**Figure 1:**
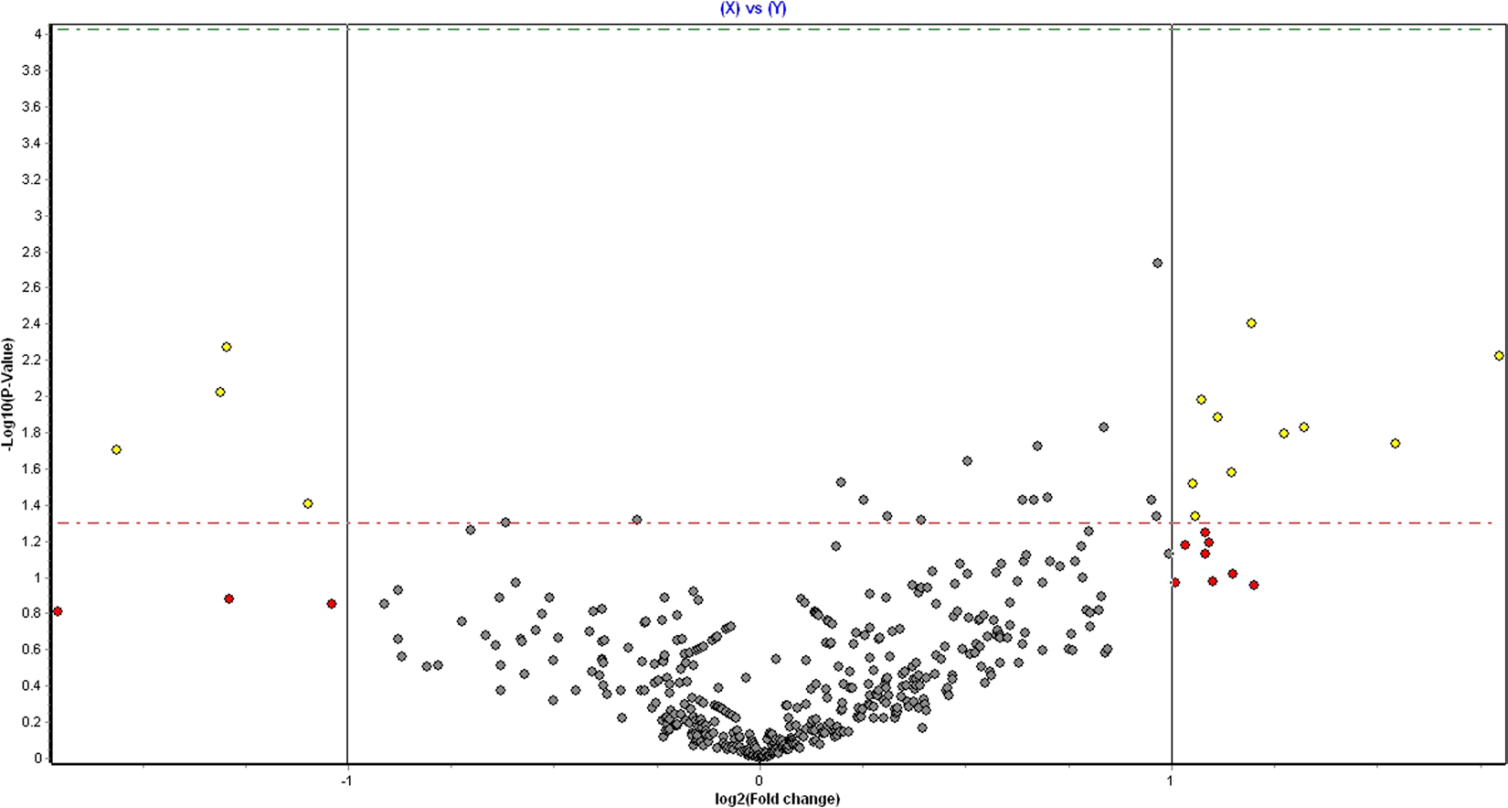
Volcano plot of microRNA detected in the follicular fluid between those women with PCOS (n = 29) versus normal women without PCOS (n = 30). Altered expression of microRNAs in a comparison between PCOS and controls are shown as fold change on the x-axis and the significance (p-value) of that fold change on the y-axis. Both are shown in log scale. Red dots: miRs with >2-fold change (+ or −) but not changing significantly. Yellow dots: miRs with >2-fold change and also changing significantly (p < 0.05).

176 miRNAs were detected, of which 29 differed significantly (Table 3, Fig. 1) between normal women and PCOS; all of the miRNA detected are shown in Supplementary Table 1.

**Table 3:** Significant differences (fold change) in the miRNA in follicular fluid between PCOS (n = 29) versus control women (n = 30).

PCOS vs Control	Fold change	P-Value
hsa-miR-381-3p	1.95136	0.0018
hsa-miR-199b-5p	2.2861	0.0040
has-miR-650	−2.45385	0.0053
hsa-miR-93-3p	3.47102	0.0060
has-miR-663b	−2.47917	0.0095
hsa-miR-361-3p	2.10148	0.0104
hsa-miR-127-3p	2.15942	0.0132
has-miR-1238-3p	1.78509	0.0149
hsa-miR-382-5p	2.4991	0.0150
hsa-miR-425-3p	2.41375	0.0161
hsa-miR-212-3p	2.91193	0.0183
hsa-miR-423-3p	1.59576	0.0189
hsa-miR-891b	−2.95618	0.0198
hsa-miR-187-3p	1.41842	0.0229
hsa-miR-513c-5p	2.21283	0.0262
hsa-miR-510-5p	1.1463	0.0300
hsa-miR-507	2.07081	0.0305
hsa-miR-509-3-5p	1.62364	0.0366
hsa-miR-638	1.18991	0.0375
hsa-miR-532-5p	1.93401	0.0375
hsa-miR-1271-5p	1.55672	0.0375
hsa-miR-193a-5p	1.58387	0.0377
hsa-miR-32-5p	−2.14067	0.0396
hsa-miR-200c-3p	2.08148	0.0459
hsa-miR-33b-3p	1.23812	0.0460
hsa-miR-675-5p	1.94931	0.0462
hsa-miR-125b-5p	1.31108	0.0481
hsa-miR-206	−1.23035	0.0486
hsa-miR-9-5p	−1.53421	0.0500

The top 7 of these (p < 0.015) were miR-381-3p, miR-199b-5p, miR-93-3p, miR-361-3p, miR-127-3p, miR-382-5p, miR-425-3p. In PCOS, miR-382-5p correlated with age and FAI, miR-199b-5p correlated with AMH and miR-93-3p correlated with C-reactive protein (CRP). In normal controls, miR-127-3p, miR382-5p and miR-425-3p correlated with the fertilisation rate; miR-127-3p correlated with insulin resistance, and miR-381-3p correlated with FAI (Table 4).

**Table 4:** Spearman’s correlation of the top 7 microRNA (p < 0.015) in follicular fluid taken at the time of oocyte retrieval during IVF in 59 age and weight matched women, 29 with PCOS and 30 normal control women.

	PCOS													
	miR-199b-5p		miR-127-3p		miR-361-3p		miR-382-5p		miR-425-3p		miR-381-3p		miR-93-3p	
	R	P-value	R	P-value	R	P-value	R	P-value	R	P-value	R	P-value	R	P-value
Age	0.348	0.095	0.215	0.29	−0.039	0.875	0.436	0.023	0.198	0.333	−0.088	0.744	−0.363	0.105
BMI	0.075	0.728	−0.091	0.658	−0.172	0.481	−0.149	0.459	−0.124	0.547	−0.088	0.745	0.04	0.862
AMH	−0.591	0.002	0.023	0.911	0.069	0.779	−0.152	0.449	−0.143	0.485	−0.073	0.789	−0.106	0.647
Fertilisation rate	0.191	0.408	0.152	0.51	−0.133	0.638	−0.095	0.674	0.023	0.92	−0.159	0.586	0.156	0.536
HOMA-IR	−0.163	0.445	−0.08	0.699	−0.244	0.314	−0.148	0.462	−0.286	0.156	−0.2	0.458	0.143	0.537
CRP	0.101	0.655	−0.056	0.796	0.221	0.393	0.247	0.233	−0.147	0.492	0.089	0.751	0.637	0.003
FAI	0.012	0.955	−0.077	0.708	−0.366	0.124	−0.419	0.03	−0.306	0.129	−0.395	0.13	−0.321	0.156
	**CONTROLS**													
	**miR-199b-5p**		**miR-127-3p**		**miR-361-3p**		**miR-382-5p**		**miR-425-3p**		**miR-381-3p**		**miR-93-3p**	
	**R**	**P-value**	**R**	**P-value**	**R**	**P-value**	**R**	**P-value**	**R**	**P-value**	**R**	**P-value**	**R**	**P-value**
Age	0.034	0.887	0.06	0.77	0.191	0.533	0.382	0.072	0.133	0.535	0.629	0.028	−0.265	0.405
BMI	0.342	0.14	−0.087	0.674	−0.41	0.164	0.142	0.517	0.167	0.435	−0.109	0.737	−0.196	0.54
AMH	−0.186	0.431	−0.168	0.413	0.022	0.943	−0.019	0.93	−0.16	0.455	0.224	0.484	-0.182	0.572
Fertilisation rate	0.395	0.104	0.449	0.024	0.119	0.712	0.537	0.012	0.424	0.049	0.436	0.18	0.073	0.83
HOMA-IR	−0.108	0.65	−0.387	0.050	0.121	0.694	0.013	0.954	−0.065	0.762	0.196	0.542	0.126	0.697
CRP	0.198	0.402	0.123	0.56	0.33	0.271	0.099	0.66	0.166	0.448	−0.209	0.537	0.333	0.29
FAI	−0.002	0.993	−0.213	0.296	−0.06	0.847	−0.308	0.153	−0.005	0.983	−0.655	0.021	0.27	0.395

BMI = body mass index; AMH = Antimullarian Hormone; HOMA-IR = insulin resistance; FAI = free androgen index; CRP = C reactive protein.

### Ingenuity pathway assessment

**Figure 2:**
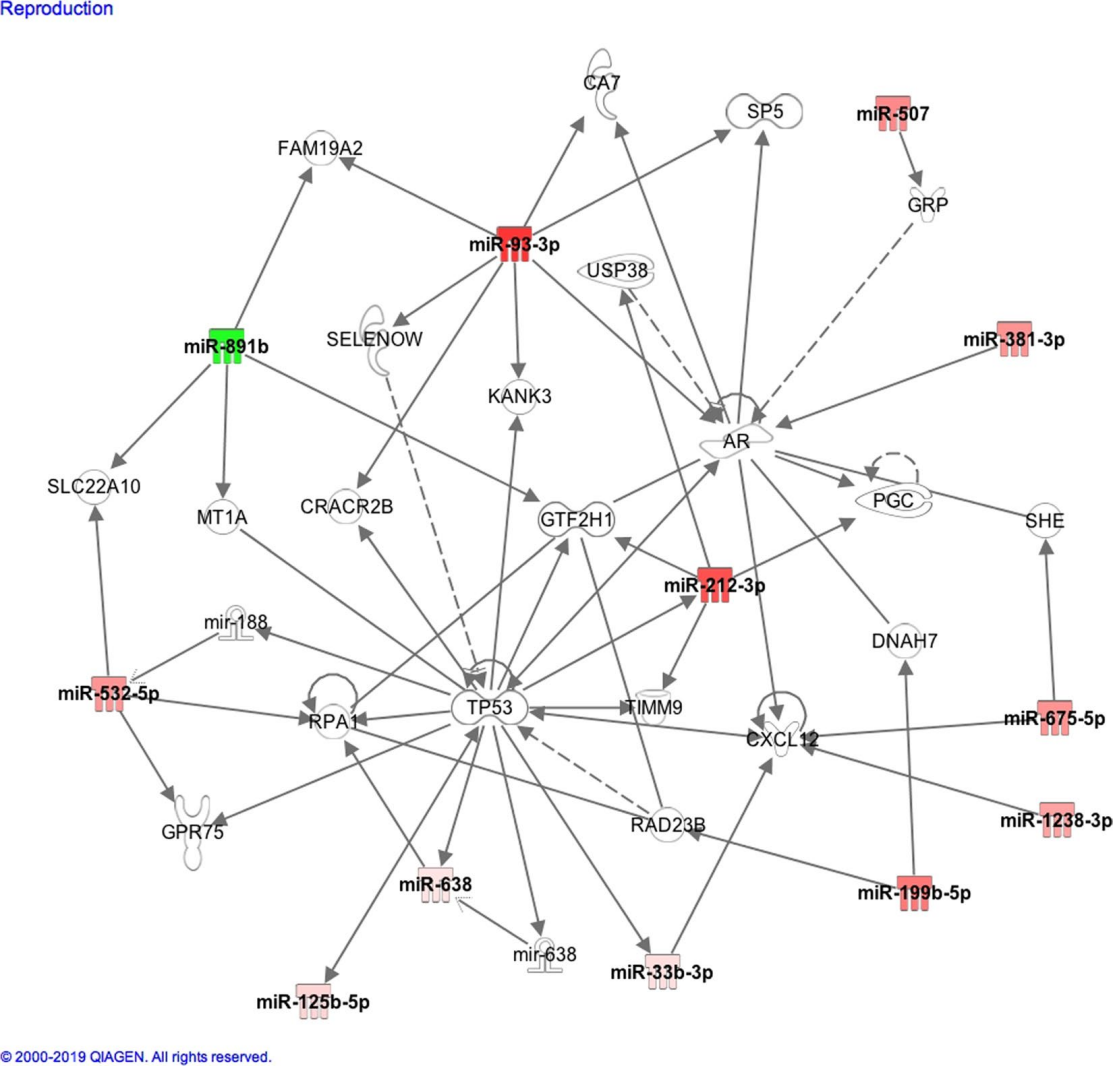
Ingenuity pathway analysis of the significantly altered miRNA found in the follicular fluid showing that 12 of these miRNA were related to the genes involved in the Organismal Injury and Abnormalities, Reproductive System Disease pathway. Strongly upregulated and downregulated miRNA are represented with dark red and green color and vice versa.

**Figure 3:**
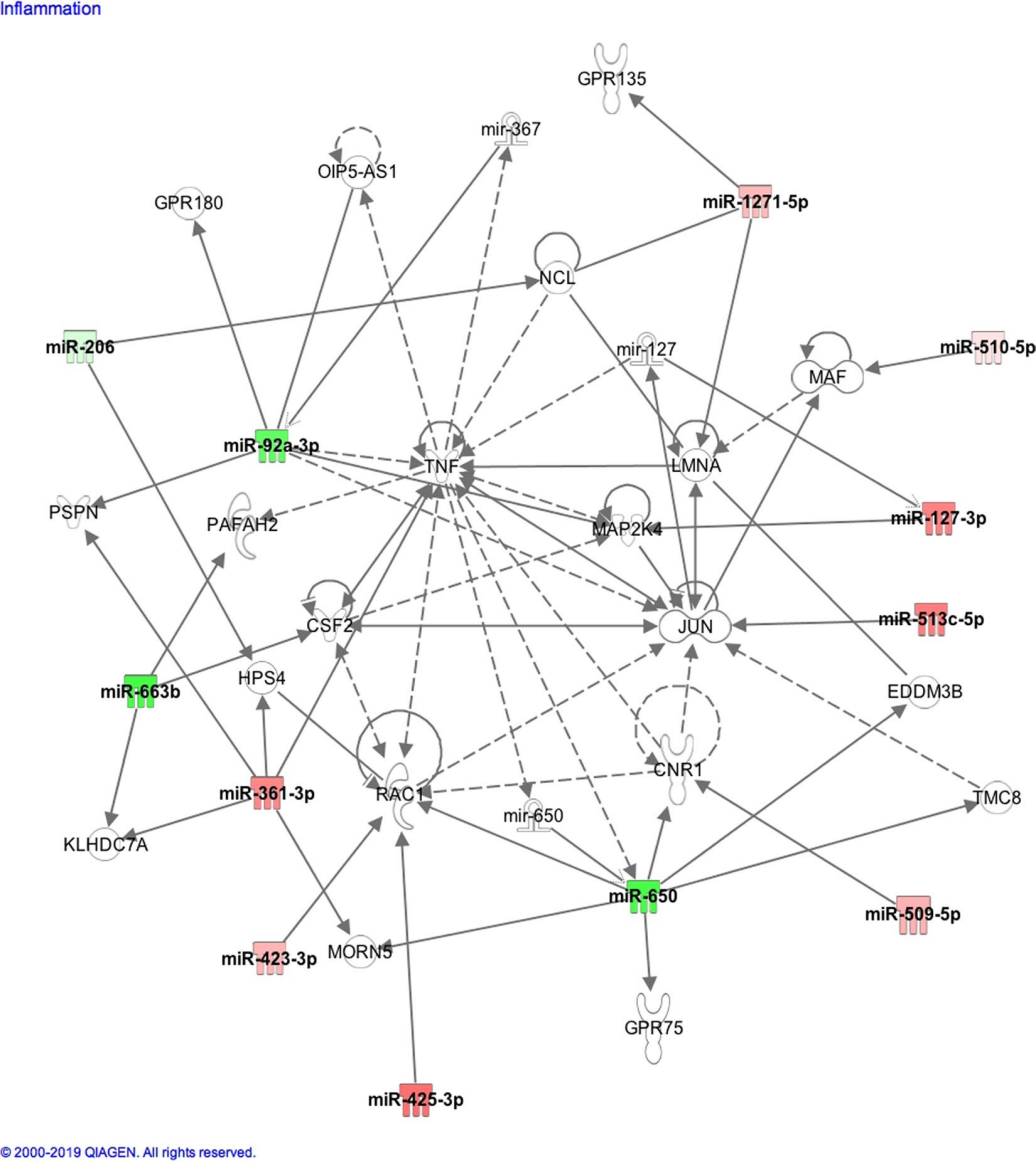
Ingenuity pathway analysis of the significantly altered miRNA found in the follicular fluid showing that 12 of these miRNA were related to the genes involved in the Inflammatory disease pathway. Strongly upregulated and downregulated miRNA are represented with dark red and green color and vice versa.

When Ingenuity pathway analysis was undertaken to look at the functional relationships, it was seen that 12 of the significantly altered miRNA related to reproductive pathways, as is shown in Fig. 2. Twelve miRNAs were related to the inflammatory disease pathway, as is shown in Fig. 3. Six miRNAs were related to the pathways implicated in benign pelvic disease: miR-212-3p, miR-32-5p, miR-200c-3p, miR-125b-5p, miR-206 and miR-9-5p.

## Discussion

Ovulatory dysfunction, increased IR, an elevated FAI and inflammation are hallmarks of PCOS and, in line with the objective, this study has shown that miRNA found in the follicular fluid were related to these parameters. In PCOS, miR-199b-5p correlated with AMH that is recognized to be elevated in PCOS and, indeed, AMH has been suggested by some studies, but not others, to be a biomarker of PCOS^29,30^ where it has a four fold association with a diagnosis of PCOS although with low sensitivity. miR-382-5p correlated with FAI that is classically elevated, and miR-93-3p correlated with C-reactive protein (CRP), a marker of inflammation, that has consistently been shown to be greater in PCOS than controls^31,32^. In addition, in normal subjects, miR-127-3p, miR-382-5p and miR-425-3p correlated with the fertilisation rate, whilst miR-127-3p correlated with IR and miR-381-3p correlated with FAI. Whether these miRNAs are involved in the associated processes would seem to be supported by the Ingenuity pathways showing the involvement of these miRNA specifically in the reproductive pathways (12 miRNAs) and inflammation (12 miRNAs). However, given that miRNA action is essentially inhibitory by inducing degradation or inhibition of protein translation^5^, their roles, either direct or indirect, require further clarification. It is recognized that miRNA may be affected by BMI^13^ and, to account for this, PCOS and normal controls were weight matched in this study; however, despite this, in normal controls, miR-1238-3p correlated with BMI.

Recent reports have indicated that dysregulated miRNAs may be key players in the ovulatory dysfunction of women with PCOS. MiRNAs have been reported to be differentially expressed in follicular fluid of women with PCOS^12,17^. Upregulated miRNAs include miRNA-9, miRNA-18b, miRNA-32, miRNA-34c and miRNA-135a, and, by analysis of target genes, these have been shown to be involved in carbohydrate metabolism, beta-cell function and steroid synthesis, so may have direct relevance to the phenotype of women with PCOS^12^. Insulin resistance is a fundamental underlying issue in PCOS; whether it is primary metabolite dysregulation in PCOS having a secondary effect such as increased free fatty acids enhancing insulin resistance^33^ or the converse that insulin resistance reflects metabolite is unclear, but miRNA may have a fundamental role such as miRNA-122 association with insulin resistance^34^, miRNA-361 is affected by a low glycemic diet that would impact on insulin resistance^35^, and miRNA-382 is involved in changes in insulin resistance with exercise^36^. MiRNAs reported to be downregulated in follicular fluid of PCOS women include miRNA-132 and miRNA-320^17^. This report was, however, discrepant with the findings of a later study which reported upregulation of miRNA-320 in follicular fluid, potentially explaining the hyperandrogenemia found in women with PCOS through downregulation of E2F1/SF-1 proteins which, in turn, cause inhibition of estradiol release into follicular fluid^18^. Therefore, despite the inconsistencies between studies which may relate to differing populations of women with varying ages^37^ or severity on the spectrum of PCOS^38^, miRNAs in follicular fluid appear to play a central role in the ovulatory dysfunction of PCOS.

Strengths of this study were the well-matched groups of women with and without PCOS, who were all undergoing *in vitro* fertilization, and the relatively large patient cohort for a study of this nature. The fact that the study was based upon follicular fluid samples as opposed to peripheral blood samples is also a major strength. Limitations were that this was a relatively small study and the women with PCOS did not have the most severe PCOS phenotype in terms of BMI and insulin resistance, and this may have reduced differences in their miRNA profiles when compared with the control women. Despite this, distinct differences were found and may be generalizable to all PCOS women.

In conclusion, miR differed in the follicular fluid between PCOS and normal control women, correlating with age, FAI, inflammation and AMH in PCOS, and with fertilization rate, insulin resistance and FAI and inflammation in control women, in accord with the Ingenuity pathway assessment.

## Supplementary Information

Supplementary Informationsyndrome. The New England journal of medicine

## Data Availability

The data that support the findings of this study are available from the corresponding author upon request.
