# Supplementary material for: Expression of microRNA in follicular fluid in women with and without PCOS
Source: Sci Rep. 2019 Nov 8;9:16306. doi: 10.1038/s41598-019-52856-5 (PMC6841741; doi:10.1038/s41598-019-52856-5)
Supplement: Supplementary file 1 — Supplementary Informationsyndrome. The New England journal of medicine [file 41598_2019_52856_MOESM1_ESM.docx]

**Supplementary Table 1.** All microRNA (176) identified and the comparison between PCOS (n=29) and control women (n=30) in the follicular fluid at oocyte retrieval during an in vitro fertilization cycle. Significant differences in miR shown in bold.

| **PCOS v control** | **Fold change** | **Difference (A-B log scale)** | **P-Value** |
| --- | --- | --- | --- |
| **hsa-miR-381-3p** | **1.95136** | **0.96448** | **0.001838849** |
| **hsa-miR-199b-5p** | **2.2861** | **1.19289** | **0.003962977** |
| **hsa-miR-93-3p** | **3.47102** | **1.79536** | **0.006028962** |
| **hsa-miR-361-3p** | **2.10148** | **1.0714** | **0.01044978** |
| **hsa-miR-127-3p** | **2.15942** | **1.11064** | **0.013174261** |
| **hsa-miR-382-5p** | **2.4991** | **1.32141** | **0.01499203** |
| **hsa-miR-425-3p** | **2.41375** | **1.27127** | **0.016114434** |
| **hsa-miR-212-3p** | **2.91193** | **1.54198** | **0.018301977** |
| **hsa-miR-423-3p** | **1.59576** | **0.67425** | **0.018914113** |
| **hsa-miR-891b** | **-2.95618** | **-1.56373** | **0.019817753** |
| **hsa-miR-187-3p** | **1.41842** | **0.50429** | **0.022928167** |
| **hsa-miR-513c-5p** | **2.21283** | **1.14589** | **0.026204145** |
| **hsa-miR-510-5p** | **1.1463** | **0.19698** | **0.030031659** |
| **hsa-miR-507** | **2.07081** | **1.05019** | **0.030484117** |
| **hsa-miR-509-3-5p** | **1.62364** | **0.69923** | **0.036574281** |
| **hsa-miR-638** | **1.18991** | **0.25085** | **0.037451875** |
| **hsa-miR-532-5p** | **1.93401** | **0.95159** | **0.037517679** |
| **hsa-miR-1271-5p** | **1.55672** | **0.63851** | **0.037535876** |
| **hsa-miR-193a-5p** | **1.58387** | **0.66345** | **0.037717575** |
| **hsa-miR-32-5p** | **-2.14067** | **-1.09807** | **0.039613694** |
| **hsa-miR-200c-3p** | **2.08148** | **1.05761** | **0.045916717** |
| **hsa-miR-33b-3p** | **1.23812** | **0.30815** | **0.046046145** |
| **hsa-miR-675-5p** | **1.94931** | **0.96297** | **0.046169162** |
| **hsa-miR-125b-5p** | **1.31108** | **0.39076** | **0.048059097** |
| **hsa-miR-206** | **-1.23035** | **-0.29907** | **0.048645736** |
| **hsa-miR-9-5p** | **-1.53421** | **-0.61749** | **0.04998321** |
| hsa-miR-18a-3p | -1.62762 | -0.70277 | 0.054737209 |
| hsa-miR-140-3p | 1.73777 | 0.79724 | 0.055786027 |
| hsa-miR-30a-3p | 2.11604 | 1.08137 | 0.056786194 |
| hsa-miR-874-3p | 2.1291 | 1.09024 | 0.064043515 |
| hsa-miR-200a-3p | 2.04573 | 1.03262 | 0.06675977 |
| hsa-miR-28-3p | 1.71592 | 0.77898 | 0.067388482 |
| hsa-miR-552-3p | 1.13593 | 0.18388 | 0.067520993 |
| hsa-miR-31-5p | 1.98916 | 0.99216 | 0.074254409 |
| hsa-miR-329-3p | 2.11679 | 1.08188 | 0.074761053 |
| hsa-miR-323a-3p | 1.56653 | 0.64758 | 0.075228781 |
| hsa-miR-214-5p | 1.55854 | 0.6402 | 0.081576881 |
| hsa-miR-106b-3p | 1.62788 | 0.70299 | 0.081915478 |
| hsa-miR-342-3p | 1.69768 | 0.76356 | 0.082001271 |
| hsa-miR-943 | 1.40058 | 0.48603 | 0.084230964 |
| hsa-miR-151a-3p | 1.50105 | 0.58597 | 0.084986231 |
| hsa-miR-19b-3p | 1.65728 | 0.72882 | 0.087154025 |
| hsa-miR-10a-5p | 1.33594 | 0.41786 | 0.092466383 |
| hsa-miR-551b-3p | 1.48711 | 0.57251 | 0.09396788 |
| hsa-miR-1247-5p | 2.21569 | 1.14776 | 0.095871579 |
| hsa-miR-539-5p | 1.41888 | 0.50475 | 0.09637249 |
| hsa-miR-195-5p | 1.72018 | 0.78256 | 0.099961405 |
| hsa-miR-574-3p | 1.5409 | 0.62378 | 0.105098561 |
| hsa-miR-223-3p | 2.14189 | 1.09889 | 0.105291586 |
| hsa-miR-16-1-3p | 2.01017 | 1.00732 | 0.106650792 |
| hsa-miR-647 | -1.51067 | -0.59519 | 0.106899794 |
| hsa-miR-191-5p | 1.61003 | 0.68709 | 0.107880079 |
| hsa-miR-125a-3p | 1.38936 | 0.47442 | 0.109563559 |
| hsa-miR-2053 | 2.29814 | 1.20047 | 0.11040877 |
| hsa-miR-21-3p | 1.29229 | 0.36993 | 0.111040082 |
| hsa-miR-24-2-5p | 1.32507 | 0.40607 | 0.114457254 |
| hsa-miR-181a-2-3p | 1.3108 | 0.39044 | 0.114595538 |
| hsa-miR-7-5p | -1.8414 | -0.88081 | 0.118567444 |
| hsa-miR-525-3p | -1.11983 | -0.16328 | 0.119794497 |
| hsa-miR-25-5p | 1.30713 | 0.3864 | 0.122676814 |
| hsa-miR-105-3p | 1.20168 | 0.26506 | 0.123565054 |
| hsa-miR-154-5p | 1.77778 | 0.83008 | 0.127008861 |
| hsa-miR-545-3p | -1.55112 | -0.63331 | 0.129090944 |
| hsa-miR-302b-3p | -1.17442 | -0.23195 | 0.129635311 |
| hsa-miR-21-5p | 1.23685 | 0.30667 | 0.130625183 |
| hsa-miR-942-5p | -1.42519 | -0.51115 | 0.131036802 |
| hsa-miR-195-3p | 1.07061 | 0.09843 | 0.1322147 |
| hsa-miR-519c-3p | 1.07117 | 0.09919 | 0.132684172 |
| hsa-miR-139-3p | -2.44398 | -1.28923 | 0.132770938 |
| hsa-miR-188-3p | -1.1087 | -0.14887 | 0.135228287 |
| hsa-miR-548k | 1.07707 | 0.10711 | 0.137931193 |
| hsa-miR-221-3p | 1.52426 | 0.60811 | 0.138955885 |
| hsa-miR-520b | 1.07857 | 0.10912 | 0.139327861 |
| hsa-miR-1539 | -2.055 | -1.03914 | 0.140267836 |
| hsa-miR-193b-3p | 1.34463 | 0.42721 | 0.14047709 |
| hsa-miR-937-3p | -1.88109 | -0.91157 | 0.140523519 |
| hsa-miR-20a-3p | -1.30425 | -0.38322 | 0.149013378 |
| hsa-miR-100-5p | 1.76864 | 0.82264 | 0.151428942 |
| hsa-miR-29a-5p | 1.7333 | 0.79352 | 0.151827762 |
| hsa-miR-1227-3p | -1.32506 | -0.40606 | 0.154452314 |
| hsa-miR-572 | -3.26286 | -1.70614 | 0.155483743 |
| hsa-miR-760 | 1.3949 | 0.48016 | 0.155594172 |
| hsa-miR-508-3p | 1.09628 | 0.13261 | 0.155862342 |
| hsa-miR-296-3p | 1.09687 | 0.13339 | 0.156390512 |
| hsa-miR-30c-2-3p | 1.09888 | 0.13604 | 0.158170706 |
| hsa-miR-106b-5p | 1.74254 | 0.80119 | 0.158386162 |
| hsa-miR-373-3p | -1.44417 | -0.53024 | 0.159396533 |
| hsa-miR-593-3p | 1.10184 | 0.13992 | 0.160727164 |
| hsa-miR-200b-5p | 1.45674 | 0.54274 | 0.161956589 |
| hsa-miR-92a-2-5p | 1.10369 | 0.14234 | 0.162289281 |
| hsa-miR-520f-3p | -1.14999 | -0.20163 | 0.162969548 |
| hsa-miR-455-3p | 1.38627 | 0.47121 | 0.16383595 |
| hsa-let-7f-1-3p | 1.42022 | 0.50612 | 0.168948926 |
| hsa-miR-324-5p | 1.44682 | 0.53288 | 0.169418439 |
| hsa-miR-193b-5p | 1.48221 | 0.56775 | 0.171895504 |
| hsa-miR-134-5p | 1.44424 | 0.53031 | 0.173862557 |
| hsa-miR-576-3p | -1.17829 | -0.23669 | 0.174157806 |
| hsa-miR-499a-5p | 1.1191 | 0.16233 | 0.174233685 |
| SNORD49A | -1.65326 | -0.72531 | 0.175733492 |
| hsa-miR-34c-5p | -1.21207 | -0.27747 | 0.175753101 |
| hsa-miR-770-5p | 1.12297 | 0.16732 | 0.176939818 |
| hsa-miR-1912 | -1.21548 | -0.28153 | 0.179610961 |
| hsa-miR-555 | 1.12859 | 0.17452 | 0.1806621 |
| hsa-miR-484 | 1.52324 | 0.60714 | 0.18555514 |
| hsa-miR-190a-5p | -1.05058 | -0.07118 | 0.187791296 |
| hsa-miR-409-3p | 1.74408 | 0.80247 | 0.187880991 |
| hsa-miR-130b-5p | -1.05447 | -0.07652 | 0.18983129 |
| hsa-miR-1203 | -1.05496 | -0.07719 | 0.190201686 |
| hsa-miR-379-5p | 1.20352 | 0.26726 | 0.190728985 |
| hsa-miR-92a-1-5p | 1.26487 | 0.33899 | 0.193114878 |
| hsa-miR-636 | -1.05998 | -0.08403 | 0.194977277 |
| hsa-miR-2110 | -1.06008 | -0.08417 | 0.195087658 |
| hsa-miR-30a-5p | 1.49052 | 0.57582 | 0.195533894 |
| hsa-miR-196b-3p | -1.45878 | -0.54476 | 0.197032692 |
| hsa-miR-513b-5p | 1.24843 | 0.32011 | 0.199042444 |
| hsa-miR-576-5p | -1.33185 | -0.41343 | 0.199774867 |
| hsa-miR-10b-5p | 1.5636 | 0.64487 | 0.20181118 |
| hsa-miR-181a-3p | 1.17479 | 0.2324 | 0.204001486 |
| hsa-miR-339-3p | 1.68861 | 0.75584 | 0.206053804 |
| hsa-miR-192-5p | -1.5872 | -0.66648 | 0.208385343 |
| hsa-miR-370-3p | 1.19388 | 0.25566 | 0.209388623 |
| hsa-miR-25-3p | 1.48932 | 0.57465 | 0.210913641 |
| hsa-miR-376b-3p | 1.49613 | 0.58123 | 0.210979585 |
| hsa-miR-671-3p | -1.07429 | -0.10339 | 0.212454932 |
| hsa-miR-376c-3p | 1.46526 | 0.55116 | 0.214022743 |
| hsa-miR-486-3p | -1.07706 | -0.1071 | 0.215883181 |
| hsa-miR-570-3p | -1.40616 | -0.49176 | 0.215980404 |
| hsa-miR-375 | 1.51606 | 0.60033 | 0.216538351 |
| hsa-miR-30d-3p | 1.22375 | 0.2913 | 0.216890537 |
| hsa-miR-514a-3p | 1.49673 | 0.58181 | 0.216929227 |
| hsa-miR-374a-5p | -1.13995 | -0.18898 | 0.218555222 |
| hsa-miR-618 | -1.84148 | -0.88086 | 0.219405332 |
| hsa-miR-941 | 1.22132 | 0.28844 | 0.219646886 |
| hsa-miR-96-3p | -1.49544 | -0.58057 | 0.220824698 |
| hsa-miR-938 | -1.29818 | -0.37649 | 0.222068931 |
| hsa-miR-96-5p | -1.14929 | -0.20074 | 0.222717094 |
| hsa-miR-566 | -1.08335 | -0.1155 | 0.223383895 |
| hsa-miR-212-5p | -1.30554 | -0.38465 | 0.22782879 |
| hsa-miR-431-5p | -1.49258 | -0.57781 | 0.228229075 |
| hsa-miR-365b-5p | 1.11642 | 0.15889 | 0.229367705 |
| hsa-miR-520g-3p | 1.12603 | 0.17124 | 0.231776775 |
| hsa-miR-101-5p | 1.12529 | 0.17029 | 0.232760445 |
| hsa-miR-432-5p | 1.17092 | 0.22764 | 0.233879425 |
| hsa-miR-215-5p | 1.55687 | 0.63865 | 0.23492246 |
| hsa-miR-29b-2-5p | 1.43826 | 0.52433 | 0.235937658 |
| hsa-miR-765 | -1.5613 | -0.64275 | 0.237015621 |
| hsa-miR-20b-5p | -1.09998 | -0.13747 | 0.240819535 |
| hsa-miR-222-3p | 1.36617 | 0.45013 | 0.241304638 |
| hsa-miR-629-5p | 1.449 | 0.53506 | 0.242963748 |
| hsa-let-7g-3p | -1.24852 | -0.32022 | 0.245910639 |
| hsa-miR-124-3p | -1.10582 | -0.14512 | 0.24609397 |
| hsa-miR-523-3p | -1.10842 | -0.1485 | 0.248302377 |
| hsa-miR-141-3p | 1.79473 | 0.84377 | 0.251253264 |
| hsa-miR-328-3p | 1.40535 | 0.49093 | 0.251470155 |
| hsa-miR-34a-5p | 1.6803 | 0.74872 | 0.251550239 |
| hsa-miR-196a-5p | -1.11377 | -0.15545 | 0.252612779 |
| hsa-miR-376a-3p | 1.69203 | 0.75876 | 0.254033816 |
| hsa-miR-125b-2-3p | 1.60853 | 0.68575 | 0.255851568 |
| hsa-miR-144-5p | -1.12565 | -0.17076 | 0.261137509 |
| hsa-miR-205-3p | 1.78724 | 0.83774 | 0.262109661 |
| hsa-miR-365a-3p | 1.43594 | 0.52199 | 0.263868137 |
| hsa-miR-133a-3p | 1.4235 | 0.50944 | 0.265252773 |
| hsa-miR-208a-3p | -1.13494 | -0.18262 | 0.266927614 |
| hsa-miR-15a-3p | -1.17535 | -0.23309 | 0.268520985 |
| hsa-miR-665 | 1.34423 | 0.42678 | 0.269591402 |
| hsa-miR-502-3p | 1.34513 | 0.42774 | 0.272103327 |
| hsa-miR-132-3p | 1.24309 | 0.31393 | 0.274584633 |
| hsa-miR-875-5p | -1.82773 | -0.87005 | 0.276909961 |
| hsa-miR-1908-5p | 1.20293 | 0.26655 | 0.281547823 |
| hsa-miR-505-3p | 1.3561 | 0.43947 | 0.285013561 |
| hsa-miR-127-5p | 1.02811 | 0.04 | 0.28570263 |
| hsa-miR-369-5p | 1.02811 | 0.04 | 0.28570263 |
| hsa-miR-491-3p | 1.02811 | 0.04 | 0.28570263 |
| hsa-miR-129-5p | 1.02811 | 0.04 | 0.28570263 |
| hsa-miR-885-5p | 1.02811 | 0.04 | 0.28570263 |
| hsa-miR-494-3p | 1.02811 | 0.04 | 0.28570263 |
| hsa-miR-211-5p | 1.02811 | 0.04 | 0.28570263 |
| hsa-miR-521 | 1.02811 | 0.04 | 0.28570263 |
| hsa-miR-608 | 1.02811 | 0.04 | 0.28570263 |
| hsa-miR-187-5p | 1.02811 | 0.04 | 0.28570263 |
| hsa-miR-449a | 1.02811 | 0.04 | 0.28570263 |
| hsa-miR-135b-5p | 1.02811 | 0.04 | 0.28570263 |
| hsa-miR-299-5p | 1.02811 | 0.04 | 0.28570263 |
| hsa-miR-490-3p | 1.02811 | 0.04 | 0.28570263 |
| hsa-miR-299-3p | 1.02811 | 0.04 | 0.28570263 |
| hsa-miR-153-3p | 1.02811 | 0.04 | 0.28570263 |
| hsa-miR-592 | 1.02811 | 0.04 | 0.28570263 |
| hsa-miR-487a-3p | 1.02811 | 0.04 | 0.28570263 |
| hsa-miR-138-1-3p | 1.02811 | 0.04 | 0.28570263 |
| hsa-miR-1244 | 1.02811 | 0.04 | 0.28570263 |
| hsa-miR-640 | 1.02811 | 0.04 | 0.28570263 |
| hsa-miR-1914-3p | 1.02811 | 0.04 | 0.28570263 |
| hsa-miR-556-5p | 1.02811 | 0.04 | 0.28570263 |
| hsa-miR-517-5p | 1.02811 | 0.04 | 0.28570263 |
| hsa-miR-558 | 1.02811 | 0.04 | 0.28570263 |
| hsa-miR-559 | 1.02811 | 0.04 | 0.28570263 |
| hsa-miR-488-5p | 1.02811 | 0.04 | 0.28570263 |
| hsa-miR-452-3p | 1.02811 | 0.04 | 0.28570263 |
| hsa-miR-200c-5p | 1.02811 | 0.04 | 0.28570263 |
| hsa-let-7a-3p | 1.02811 | 0.04 | 0.28570263 |
| hsa-miR-135a-3p | 1.02811 | 0.04 | 0.28570263 |
| hsa-miR-744-3p | 1.02811 | 0.04 | 0.28570263 |
| hsa-miR-1248 | 1.02811 | 0.04 | 0.28570263 |
| hsa-miR-330-5p | 1.02811 | 0.04 | 0.28570263 |
| hsa-miR-340-3p | 1.02811 | 0.04 | 0.28570263 |
| hsa-miR-34a-3p | 1.02811 | 0.04 | 0.28570263 |
| hsa-miR-1270 | 1.02811 | 0.04 | 0.28570263 |
| hsa-miR-1200 | 1.02811 | 0.04 | 0.28570263 |
| hsa-miR-34b-5p | 1.02811 | 0.04 | 0.28570263 |
| hsa-miR-561-3p | 1.02811 | 0.04 | 0.28570263 |
| hsa-miR-526b-3p | 1.02811 | 0.04 | 0.28570263 |
| hsa-miR-1911-3p | 1.02811 | 0.04 | 0.28570263 |
| hsa-miR-562 | 1.02811 | 0.04 | 0.28570263 |
| hsa-miR-200a-5p | 1.02811 | 0.04 | 0.28570263 |
| hsa-miR-944 | 1.02811 | 0.04 | 0.28570263 |
| hsa-miR-374b-5p | -1.30541 | -0.38451 | 0.286026816 |
| hsa-miR-506-3p | 1.07929 | 0.11008 | 0.286622307 |
| hsa-miR-138-5p | -1.178 | -0.23633 | 0.286650833 |
| hsa-let-7d-5p | -1.30486 | -0.38389 | 0.287171063 |
| hsa-miR-450b-5p | -1.41591 | -0.50173 | 0.29025225 |
| hsa-miR-550a-3p | -1.17622 | -0.23416 | 0.291547186 |
| hsa-miR-516a-3p | -1.21919 | -0.28592 | 0.292124308 |
| hsa-miR-30e-5p | 1.4979 | 0.58294 | 0.296125438 |
| hsa-miR-224-3p | 1.54648 | 0.62899 | 0.29644466 |
| hsa-let-7i-3p | -1.30332 | -0.38219 | 0.29764448 |
| hsa-miR-1538 | -1.13302 | -0.18017 | 0.299144021 |
| mmu-miR-378a-3p | 1.30079 | 0.37939 | 0.300067622 |
| hsa-miR-302a-3p | -1.19452 | -0.25644 | 0.30285802 |
| hsa-miR-483-3p | -1.71842 | -0.78108 | 0.308786958 |
| hsa-miR-503-5p | -1.54715 | -0.62961 | 0.309460467 |
| hsa-miR-643 | -1.11888 | -0.16206 | 0.30966135 |
| hsa-miR-1972 | 1.45193 | 0.53797 | 0.310253894 |
| hsa-miR-551a | 1.29306 | 0.37079 | 0.311737528 |
| hsa-miR-409-5p | 1.14081 | 0.19005 | 0.313518269 |
| hsa-miR-136-5p | -1.7511 | -0.80826 | 0.313818493 |
| hsa-miR-155-5p | -1.14339 | -0.19332 | 0.324247673 |
| hsa-miR-342-5p | 1.2097 | 0.27465 | 0.32730076 |
| hsa-miR-142-5p | 1.47278 | 0.55854 | 0.3311289 |
| hsa-miR-181b-5p | 1.27727 | 0.35306 | 0.333319171 |
| hsa-miR-300 | 1.16297 | 0.21782 | 0.333503201 |
| hsa-miR-520d-3p | -1.32649 | -0.40762 | 0.334255764 |
| hsa-miR-122-5p | -1.48585 | -0.57129 | 0.343659109 |
| hsa-miR-338-3p | 1.34266 | 0.42509 | 0.344154563 |
| hsa-miR-518f-5p | 1.27194 | 0.34703 | 0.345625016 |
| hsa-miR-505-5p | -1.31045 | -0.39006 | 0.348600414 |
| hsa-miR-369-3p | 1.30537 | 0.38445 | 0.349411837 |
| hsa-miR-200b-3p | 1.38261 | 0.4674 | 0.350933093 |
| hsa-miR-769-5p | 1.47579 | 0.56149 | 0.352011632 |
| hsa-miR-20b-3p | -1.02419 | -0.03448 | 0.358081879 |
| hsa-miR-301b | -1.02419 | -0.03448 | 0.358081879 |
| hsa-miR-550a-5p | -1.02419 | -0.03448 | 0.358081879 |
| hsa-miR-518c-3p | -1.02419 | -0.03448 | 0.358081879 |
| hsa-miR-367-3p | -1.02419 | -0.03448 | 0.358081879 |
| hsa-miR-625-3p | -1.02419 | -0.03448 | 0.358081879 |
| hsa-miR-302d-3p | -1.02419 | -0.03448 | 0.358081879 |
| hsa-miR-362-5p | -1.02419 | -0.03448 | 0.358081879 |
| hsa-miR-224-5p | -1.02419 | -0.03448 | 0.358081879 |
| hsa-miR-302c-5p | -1.02419 | -0.03448 | 0.358081879 |
| hsa-miR-662 | -1.02419 | -0.03448 | 0.358081879 |
| hsa-miR-181c-5p | -1.02419 | -0.03448 | 0.358081879 |
| hsa-miR-595 | -1.02419 | -0.03448 | 0.358081879 |
| hsa-miR-34b-3p | -1.02419 | -0.03448 | 0.358081879 |
| hsa-miR-502-5p | -1.02419 | -0.03448 | 0.358081879 |
| hsa-miR-216a-5p | -1.02419 | -0.03448 | 0.358081879 |
| hsa-miR-583 | -1.02419 | -0.03448 | 0.358081879 |
| hsa-miR-620 | -1.02419 | -0.03448 | 0.358081879 |
| hsa-miR-522-3p | -1.02419 | -0.03448 | 0.358081879 |
| hsa-miR-450b-3p | -1.02419 | -0.03448 | 0.358081879 |
| hsa-miR-27b-5p | -1.02419 | -0.03448 | 0.358081879 |
| hsa-miR-585-3p | -1.02419 | -0.03448 | 0.358081879 |
| hsa-miR-302f | -1.02419 | -0.03448 | 0.358081879 |
| hsa-miR-769-3p | -1.02419 | -0.03448 | 0.358081879 |
| hsa-miR-221-5p | -1.02419 | -0.03448 | 0.358081879 |
| hsa-miR-19a-5p | -1.02419 | -0.03448 | 0.358081879 |
| hsa-miR-1205 | -1.02419 | -0.03448 | 0.358081879 |
| hsa-miR-544a | -1.02419 | -0.03448 | 0.358081879 |
| hsa-miR-767-5p | -1.02419 | -0.03448 | 0.358081879 |
| hsa-miR-129-1-3p | -1.02419 | -0.03448 | 0.358081879 |
| hsa-miR-610 | -1.02419 | -0.03448 | 0.358081879 |
| hsa-miR-548j-5p | -1.02419 | -0.03448 | 0.358081879 |
| hsa-miR-1255b-5p | -1.02419 | -0.03448 | 0.358081879 |
| hsa-miR-556-3p | -1.02419 | -0.03448 | 0.358081879 |
| hsa-miR-614 | -1.02419 | -0.03448 | 0.358081879 |
| hsa-miR-208b-3p | -1.02419 | -0.03448 | 0.358081879 |
| hsa-miR-367-5p | -1.02419 | -0.03448 | 0.358081879 |
| hsa-miR-302e | -1.02419 | -0.03448 | 0.358081879 |
| hsa-miR-1206 | -1.02419 | -0.03448 | 0.358081879 |
| hsa-miR-92b-5p | -1.02419 | -0.03448 | 0.358081879 |
| hsa-miR-600 | -1.02419 | -0.03448 | 0.358081879 |
| hsa-miR-183-3p | -1.02419 | -0.03448 | 0.358081879 |
| hsa-miR-579-3p | -1.02419 | -0.03448 | 0.358081879 |
| hsa-miR-320d | 1.23962 | 0.3099 | 0.35913295 |
| hsa-miR-135a-5p | -1.17051 | -0.22714 | 0.360535446 |
| hsa-miR-532-3p | 1.32138 | 0.40205 | 0.362361346 |
| hsa-miR-543 | 1.38265 | 0.46743 | 0.364187083 |
| hsa-miR-873-5p | 1.29465 | 0.37256 | 0.366339592 |
| hsa-miR-376a-5p | 1.23758 | 0.30752 | 0.36671366 |
| hsa-miR-218-5p | 1.29926 | 0.37769 | 0.369182181 |
| hsa-miR-1 | -1.18794 | -0.24846 | 0.370168326 |
| hsa-miR-488-3p | -1.13006 | -0.17639 | 0.375845854 |
| hsa-let-7b-3p | 1.46057 | 0.54653 | 0.382540138 |
| hsa-miR-876-3p | -1.14556 | -0.19605 | 0.383792524 |
| hsa-miR-152-3p | -1.19466 | -0.2566 | 0.38437893 |
| hsa-miR-582-5p | 1.23298 | 0.30215 | 0.391575671 |
| hsa-miR-183-5p | -1.16506 | -0.22041 | 0.392048237 |
| hsa-miR-627-5p | 1.14992 | 0.20153 | 0.392587559 |
| hsa-miR-99a-5p | 1.09868 | 0.13577 | 0.393158001 |
| hsa-miR-424-3p | 1.19971 | 0.26269 | 0.393205607 |
| hsa-miR-411-5p | 1.30829 | 0.38768 | 0.394660917 |
| hsa-miR-1260a | -1.30338 | -0.38225 | 0.39676853 |
| hsa-miR-654-3p | 1.3007 | 0.37929 | 0.397617284 |
| hsa-miR-335-5p | 1.27989 | 0.35602 | 0.39921094 |
| hsa-miR-513a-3p | 1.27116 | 0.34614 | 0.403194887 |
| hsa-miR-22-5p | 1.37151 | 0.45576 | 0.406807156 |
| hsa-miR-19a-3p | 1.23611 | 0.30581 | 0.408097834 |
| hsa-miR-92a-3p | 1.1675 | 0.22342 | 0.408101677 |
| hsa-miR-184 | -1.07312 | -0.10181 | 0.408133152 |
| hsa-miR-125a-5p | 1.16261 | 0.21737 | 0.409092448 |
| hsa-miR-675-3p | 1.29665 | 0.37479 | 0.413034734 |
| hsa-miR-182-3p | 1.09027 | 0.12469 | 0.416308674 |
| hsa-miR-23a-3p | 1.11733 | 0.16005 | 0.419482149 |
| hsa-miR-424-5p | -1.21598 | -0.28212 | 0.419788348 |
| hsa-miR-26a-5p | -1.2645 | -0.33857 | 0.420934 |
| hsa-miR-483-5p | 1.36925 | 0.45339 | 0.421700653 |
| hsa-miR-107 | -1.36465 | -0.44853 | 0.42218841 |
| hsa-miR-26b-5p | -1.22318 | -0.29064 | 0.423519563 |
| hsa-miR-24-3p | 1.21973 | 0.28656 | 0.424474656 |
| U6 snRNA | -1.54852 | -0.63089 | 0.425610064 |
| hsa-miR-500a-5p | -1.16568 | -0.22117 | 0.437882463 |
| hsa-miR-145-5p | 1.21548 | 0.28153 | 0.44261757 |
| hsa-miR-652-3p | -1.29328 | -0.37104 | 0.447044516 |
| hsa-miR-199a-3p | 1.20317 | 0.26684 | 0.447849036 |
| hsa-miR-136-3p | 1.37243 | 0.45674 | 0.44920118 |
| hsa-miR-23b-5p | 1.24025 | 0.31064 | 0.452979922 |
| hsa-miR-214-3p | 1.21876 | 0.28542 | 0.453328838 |
| hsa-miR-30e-3p | 1.26792 | 0.34246 | 0.457744512 |
| hsa-miR-320b | 1.11963 | 0.16302 | 0.46483672 |
| hsa-miR-616-3p | -1.12132 | -0.1652 | 0.464898267 |
| hsa-miR-10a-3p | 1.31798 | 0.39833 | 0.470666064 |
| hsa-miR-511-5p | -1.10644 | -0.14592 | 0.477428031 |
| hsa-miR-624-5p | -1.41637 | -0.5022 | 0.47777586 |
| hsa-miR-509-3p | 1.23103 | 0.29987 | 0.481143032 |
| hsa-miR-501-3p | 1.29391 | 0.37174 | 0.488306666 |
| hsa-miR-106a-5p | 1.2823 | 0.35873 | 0.488586055 |
| hsa-miR-202-5p | 1.23655 | 0.30632 | 0.494090494 |
| hsa-miR-1207-5p | -1.1908 | -0.25193 | 0.4960589 |
| hsa-miR-1185-5p | 1.14827 | 0.19946 | 0.496293911 |
| hsa-miR-10b-3p | -1.10065 | -0.13836 | 0.4967646 |
| hsa-miR-33a-5p | 1.31963 | 0.40013 | 0.503317122 |
| hsa-miR-320a | 1.07995 | 0.11097 | 0.503326027 |
| hsa-miR-423-5p | -1.1353 | -0.18307 | 0.503625048 |
| hsa-miR-331-3p | 1.2015 | 0.26484 | 0.505850176 |
| hsa-miR-146b-3p | -1.07932 | -0.11012 | 0.510250295 |
| hsa-miR-876-5p | 1.04508 | 0.06362 | 0.512850006 |
| hsa-miR-151a-5p | 1.20391 | 0.26773 | 0.513755707 |
| hsa-miR-548b-3p | 1.04613 | 0.06506 | 0.51604085 |
| hsa-miR-877-5p | 1.21071 | 0.27585 | 0.518483779 |
| hsa-let-7e-3p | -1.07403 | -0.10304 | 0.518909076 |
| SNORD38B | 1.28443 | 0.36113 | 0.519926619 |
| hsa-miR-125b-1-3p | -1.07325 | -0.10199 | 0.520268815 |
| hsa-miR-934 | -1.07249 | -0.10097 | 0.521619445 |
| hsa-miR-26b-3p | 1.0651 | 0.09098 | 0.525027899 |
| hsa-miR-126-3p | 1.25979 | 0.33318 | 0.525099152 |
| hsa-miR-1265 | -1.06924 | -0.09658 | 0.527658836 |
| hsa-miR-301a-3p | -1.20036 | -0.26347 | 0.528419178 |
| hsa-miR-128-3p | 1.17976 | 0.23849 | 0.529633838 |
| hsa-miR-150-5p | 1.306 | 0.38516 | 0.532322415 |
| hsa-miR-655-3p | -1.12256 | -0.16679 | 0.534060778 |
| hsa-miR-146a-5p | 1.18893 | 0.24966 | 0.534836373 |
| hsa-miR-339-5p | 1.25559 | 0.32837 | 0.534970613 |
| hsa-miR-1181 | -1.06527 | -0.09122 | 0.535660707 |
| hsa-miR-202-3p | 1.14823 | 0.19941 | 0.536714555 |
| hsa-miR-940 | 1.3212 | 0.40185 | 0.543759546 |
| hsa-miR-181c-3p | -1.16254 | -0.21728 | 0.547039413 |
| hsa-miR-130b-3p | 1.14689 | 0.19772 | 0.547256331 |
| hsa-miR-15b-3p | 1.25905 | 0.33234 | 0.550272395 |
| hsa-miR-629-3p | -1.05884 | -0.08249 | 0.550313653 |
| hsa-miR-18b-5p | 1.25765 | 0.33073 | 0.562317242 |
| hsa-miR-29c-5p | -1.05206 | -0.07322 | 0.568592543 |
| hsa-miR-708-5p | -1.14345 | -0.19339 | 0.570657842 |
| hsa-miR-95-3p | -1.15494 | -0.20781 | 0.575866222 |
| hsa-miR-922 | -1.04762 | -0.06711 | 0.582515005 |
| hsa-miR-935 | -1.11992 | -0.1634 | 0.587302974 |
| hsa-miR-139-5p | 1.17863 | 0.23711 | 0.590350994 |
| hsa-miR-130a-3p | -1.16927 | -0.22561 | 0.592017796 |
| hsa-miR-450a-5p | 1.18566 | 0.24569 | 0.593000133 |
| hsa-miR-590-5p | -1.17268 | -0.22981 | 0.596388327 |
| hsa-miR-16-5p | 1.23076 | 0.29955 | 0.597546729 |
| hsa-miR-1179 | -1.17265 | -0.22977 | 0.598064457 |
| hsa-miR-671-5p | -1.26286 | -0.3367 | 0.600392584 |
| hsa-miR-487b-3p | 1.04905 | 0.06908 | 0.600842051 |
| hsa-miR-1269a | 1.21092 | 0.27611 | 0.601193172 |
| hsa-miR-496 | -1.04212 | -0.05953 | 0.60237907 |
| hsa-miR-24-1-5p | 1.1801 | 0.23891 | 0.603243766 |
| hsa-miR-663a | 1.25573 | 0.32853 | 0.604933543 |
| hsa-miR-29a-3p | 1.09759 | 0.13435 | 0.605429123 |
| hsa-miR-33b-5p | -1.10522 | -0.14433 | 0.609326122 |
| hsa-miR-766-3p | -1.16484 | -0.22013 | 0.614367429 |
| hsa-miR-126-5p | -1.11493 | -0.15695 | 0.615447101 |
| hsa-miR-1911-5p | -1.10661 | -0.14615 | 0.617170776 |
| hsa-miR-29c-3p | -1.13306 | -0.18022 | 0.61990128 |
| hsa-miR-30d-5p | 1.09116 | 0.12586 | 0.620329866 |
| hsa-miR-133b | -1.17929 | -0.23792 | 0.621300706 |
| hsa-miR-33a-3p | -1.0785 | -0.10903 | 0.63383723 |
| hsa-miR-145-3p | 1.12382 | 0.16841 | 0.641012306 |
| hsa-miR-101-3p | -1.12694 | -0.17242 | 0.643913833 |
| hsa-miR-433-3p | 1.09026 | 0.12467 | 0.64541302 |
| hsa-miR-1237-3p | -1.10248 | -0.14075 | 0.64597429 |
| hsa-miR-99b-5p | -1.16851 | -0.22467 | 0.646396701 |
| hsa-miR-452-5p | -1.14899 | -0.20036 | 0.648973696 |
| hsa-miR-1468-5p | 1.06202 | 0.08681 | 0.660492859 |
| hsa-let-7g-5p | -1.15252 | -0.20479 | 0.663538063 |
| hsa-miR-345-5p | -1.15115 | -0.20308 | 0.663602467 |
| hsa-miR-761 | 1.11974 | 0.16316 | 0.665653251 |
| hsa-miR-30c-5p | -1.09617 | -0.13247 | 0.665721039 |
| hsa-miR-188-5p | 1.05009 | 0.07052 | 0.667074509 |
| hsa-miR-28-5p | 1.13757 | 0.18595 | 0.669861799 |
| hsa-miR-7-2-3p | -1.1101 | -0.15069 | 0.672467318 |
| hsa-miR-486-5p | 1.31464 | 0.39466 | 0.677391026 |
| hsa-miR-22-3p | -1.1015 | -0.13947 | 0.681648068 |
| hsa-let-7c-5p | 1.10778 | 0.14767 | 0.687132481 |
| hsa-miR-320c | 1.08067 | 0.11192 | 0.696213829 |
| hsa-miR-382-3p | -1.16426 | -0.21942 | 0.698212926 |
| hsa-miR-149-5p | -1.09559 | -0.13171 | 0.699290444 |
| hsa-let-7f-5p | 1.13667 | 0.18482 | 0.700603754 |
| hsa-miR-182-5p | -1.03972 | -0.0562 | 0.703809275 |
| hsa-miR-887-3p | 1.0967 | 0.13316 | 0.706478774 |
| hsa-miR-485-3p | 1.09187 | 0.1268 | 0.70658521 |
| hsa-miR-645 | -1.17152 | -0.22838 | 0.707147049 |
| hsa-miR-103a-3p | -1.16751 | -0.22343 | 0.707803367 |
| hsa-miR-20a-5p | 1.16021 | 0.21439 | 0.711034064 |
| hsa-miR-205-5p | 1.15015 | 0.20183 | 0.71434764 |
| hsa-miR-495-3p | 1.13176 | 0.17857 | 0.715016178 |
| hsa-miR-140-5p | 1.11291 | 0.15433 | 0.715666087 |
| hsa-let-7f-2-3p | 1.1429 | 0.1927 | 0.716718323 |
| hsa-miR-148a-3p | -1.08186 | -0.11352 | 0.723244058 |
| hsa-miR-15a-5p | -1.11316 | -0.15466 | 0.724181891 |
| hsa-miR-602 | -1.12223 | -0.16637 | 0.725549396 |
| hsa-miR-17-5p | 1.10991 | 0.15045 | 0.725604446 |
| hsa-miR-149-3p | -1.04595 | -0.06482 | 0.727562246 |
| hsa-miR-1256 | 1.01573 | 0.02251 | 0.728918811 |
| hsa-miR-29b-3p | 1.11415 | 0.15594 | 0.73124812 |
| hsa-miR-889-3p | 1.01536 | 0.02199 | 0.733384099 |
| hsa-miR-616-5p | 1.02089 | 0.02983 | 0.740288957 |
| hsa-miR-337-5p | 1.01944 | 0.02778 | 0.74040849 |
| hsa-miR-324-3p | 1.06792 | 0.0948 | 0.742463791 |
| hsa-miR-362-3p | -1.09448 | -0.13025 | 0.74340537 |
| hsa-miR-659-3p | -1.01857 | -0.02655 | 0.743745799 |
| hsa-miR-18a-5p | -1.12144 | -0.16536 | 0.748903799 |
| hsa-miR-31-3p | 1.13807 | 0.18659 | 0.750416667 |
| hsa-miR-378a-5p | -1.08929 | -0.12339 | 0.751678195 |
| hsa-let-7a-5p | -1.1111 | -0.15199 | 0.753783131 |
| hsa-miR-639 | -1.06463 | -0.09036 | 0.759589762 |
| hsa-miR-361-5p | 1.07468 | 0.10391 | 0.75989755 |
| hsa-miR-548e-3p | -1.03433 | -0.0487 | 0.760425151 |
| hsa-miR-335-3p | -1.17753 | -0.23576 | 0.761919093 |
| hsa-miR-542-5p | 1.13481 | 0.18245 | 0.763562786 |
| hsa-miR-412-3p | -1.03537 | -0.05014 | 0.769645356 |
| hsa-miR-421 | 1.05303 | 0.07455 | 0.780032845 |
| hsa-miR-323a-5p | 1.01167 | 0.01674 | 0.782472695 |
| hsa-miR-454-3p | 1.02662 | 0.0379 | 0.786104589 |
| hsa-miR-1909-3p | -1.04319 | -0.06101 | 0.786775017 |
| hsa-miR-203a | -1.11207 | -0.15325 | 0.790040176 |
| hsa-miR-920 | 1.03273 | 0.04646 | 0.792646958 |
| hsa-miR-17-3p | -1.11417 | -0.15597 | 0.798901859 |
| hsa-miR-497-5p | 1.09828 | 0.13525 | 0.799224082 |
| hsa-miR-380-5p | 1.05475 | 0.07691 | 0.801150676 |
| hsa-miR-377-3p | 1.054 | 0.07588 | 0.80154501 |
| hsa-miR-429 | 1.05091 | 0.07164 | 0.802425446 |
| hsa-miR-525-5p | -1.01264 | -0.01812 | 0.804493307 |
| hsa-miR-1253 | 1.03851 | 0.05451 | 0.805289252 |
| hsa-miR-363-3p | -1.10386 | -0.14256 | 0.81447968 |
| hsa-miR-185-5p | 1.09467 | 0.1305 | 0.814771825 |
| hsa-miR-744-5p | -1.01495 | -0.02141 | 0.815487022 |
| hsa-miR-582-3p | -1.09005 | -0.12439 | 0.818179046 |
| hsa-miR-660-5p | -1.06382 | -0.08926 | 0.82316336 |
| hsa-miR-493-3p | 1.03648 | 0.05169 | 0.824036908 |
| hsa-miR-597-5p | 1.05526 | 0.0776 | 0.824845635 |
| hsa-miR-23b-3p | 1.02556 | 0.03641 | 0.831326721 |
| hsa-miR-635 | -1.01042 | -0.01495 | 0.831483303 |
| hsa-miR-142-3p | 1.10518 | 0.14428 | 0.83387736 |
| hsa-miR-340-5p | 1.04324 | 0.06107 | 0.834871435 |
| hsa-miR-455-5p | 1.0618 | 0.08651 | 0.840348212 |
| hsa-miR-410-3p | 1.02542 | 0.03622 | 0.84139822 |
| hsa-miR-143-5p | -1.06464 | -0.09036 | 0.844260823 |
| hsa-miR-377-5p | 1.0586 | 0.08216 | 0.84522378 |
| hsa-miR-877-3p | -1.00928 | -0.01332 | 0.846396674 |
| hsa-miR-27a-3p | -1.05955 | -0.08346 | 0.847028125 |
| hsa-miR-1296-5p | 1.04893 | 0.06892 | 0.847735308 |
| hsa-miR-196b-5p | 1.02106 | 0.03007 | 0.849336706 |
| hsa-miR-451a | -1.118 | -0.16092 | 0.851156417 |
| hsa-let-7e-5p | 1.04994 | 0.0703 | 0.85250478 |
| hsa-miR-7-1-3p | -1.03853 | -0.05454 | 0.853538812 |
| hsa-miR-598-3p | -1.04859 | -0.06844 | 0.854219737 |
| hsa-miR-577 | -1.10033 | -0.13794 | 0.85719342 |
| hsa-miR-30b-5p | -1.04755 | -0.06702 | 0.860154502 |
| hsa-miR-615-3p | -1.03511 | -0.04978 | 0.863207018 |
| hsa-miR-132-5p | -1.05423 | -0.07619 | 0.866637312 |
| hsa-miR-99b-3p | 1.06669 | 0.09315 | 0.868753652 |
| hsa-miR-210-3p | 1.03941 | 0.05576 | 0.872419736 |
| hsa-miR-501-5p | -1.07524 | -0.10466 | 0.87450541 |
| hsa-miR-143-3p | 1.04501 | 0.06352 | 0.875321852 |
| hsa-miR-219a-5p | 1.04584 | 0.06467 | 0.882272833 |
| hsa-miR-654-5p | 1.02614 | 0.03722 | 0.882785063 |
| hsa-miR-98-5p | 1.03734 | 0.05289 | 0.884357205 |
| hsa-miR-548c-5p | 1.06983 | 0.09738 | 0.884456854 |
| hsa-miR-589-5p | -1.00647 | -0.00931 | 0.886358983 |
| hsa-miR-491-5p | -1.05718 | -0.08022 | 0.887420698 |
| hsa-let-7i-5p | 1.04405 | 0.0622 | 0.887465966 |
| hsa-miR-326 | 1.04823 | 0.06795 | 0.887534929 |
| hsa-miR-16-2-3p | -1.04527 | -0.06387 | 0.88931766 |
| hsa-miR-499a-3p | 1.02701 | 0.03845 | 0.897454727 |
| hsa-let-7b-5p | -1.02557 | -0.03642 | 0.908396955 |
| hsa-miR-186-5p | -1.02151 | -0.0307 | 0.913684629 |
| hsa-miR-589-3p | -1.03175 | -0.04509 | 0.920353273 |
| hsa-miR-508-5p | 1.00712 | 0.01023 | 0.921756447 |
| hsa-miR-92b-3p | 1.02485 | 0.03541 | 0.922294567 |
| hsa-miR-379-3p | 1.02888 | 0.04107 | 0.923975976 |
| hsa-miR-664a-3p | -1.01122 | -0.0161 | 0.925046284 |
| hsa-miR-27b-3p | -1.01742 | -0.02492 | 0.925150885 |
| hsa-miR-148b-3p | 1.01981 | 0.02831 | 0.930537043 |
| hsa-miR-651-5p | -1.0195 | -0.02787 | 0.932886812 |
| hsa-miR-337-3p | 1.01039 | 0.01491 | 0.934230398 |
| hsa-miR-296-5p | 1.02182 | 0.03114 | 0.93826366 |
| hsa-miR-15b-5p | 1.03038 | 0.04317 | 0.939456763 |
| hsa-miR-425-5p | 1.01898 | 0.02713 | 0.94374717 |
| hsa-miR-181a-5p | -1.02103 | -0.03002 | 0.950551174 |
| hsa-miR-1537-3p | -1.02189 | -0.03125 | 0.957017559 |
| hsa-let-7d-3p | 1.00632 | 0.0091 | 0.963000251 |
| hsa-miR-548a-3p | -1.01271 | -0.01822 | 0.96536853 |
| hsa-miR-93-5p | 1.01578 | 0.02259 | 0.966061175 |
| hsa-miR-197-3p | 1.01411 | 0.02022 | 0.968631613 |
| hsa-miR-141-5p | -1.01249 | -0.01791 | 0.969710429 |
| hsa-miR-146b-5p | -1.00508 | -0.00731 | 0.970740962 |
| hsa-miR-584-5p | -1.01132 | -0.01624 | 0.971926585 |
| hsa-miR-642a-5p | -1.01117 | -0.01603 | 0.972068787 |
| hsa-miR-628-3p | -1.00251 | -0.00362 | 0.973009282 |
| hsa-miR-144-3p | -1.01978 | -0.02826 | 0.974342047 |
| hsa-miR-99a-3p | 1.01124 | 0.01612 | 0.978216656 |
| hsa-miR-194-5p | -1.00728 | -0.01046 | 0.984981463 |
| hsa-miR-223-5p | 1.00464 | 0.00668 | 0.98670467 |
| hsa-miR-632 | 1.00385 | 0.00555 | 0.989538558 |
| hsa-miR-490-5p | 1.00044 | 0.00063 | > 0.99 |
| hsa-miR-137 | -1.00085 | -0.00123 | > 0.99 |
| hsa-miR-431-3p | 1. | 0. | > 0.99 |
|  |  |  |  |
